# Reduced Diversity in the Bacteriome of the Phytophagous Mite *Brevipalpus yothersi* (Acari: Tenuipalpidae)

**DOI:** 10.3390/insects7040080

**Published:** 2016-12-20

**Authors:** Oscar E. Ospina, Steven E. Massey, Jose Carlos Verle Rodrigues

**Affiliations:** 1 Center for Excellence in Quarantine and Invasive Species, Agricultural Experimental Station-Río Piedras, Crops and Agro-Environmental Sciences Department, University of Puerto Rico-Mayaguez, 1193 Calle Guayacán, San Juan, PR 00926-1118, USA; ospina.oe@gmail.com; 2 Bioinformatics Laboratory, Department of Biology, University of Puerto Rico-Río Piedras, San Juan, PR 00931-3360, USA; stevenemassey@gmail.com

**Keywords:** bacterial diversity, *Brevipalpus yothersi*, false spider mite, *Cardinium*, habitat filtering, 16S ribosomal RNA, Tetranychoidea, *Raoiella indica*, *Tetranychus evansi*, *Oligonychus*

## Abstract

Tenuipalpidae comprises mites that transmit viruses to agriculturally important plants. Several tenuipalpid species present parthenogenesis, and in *Brevipalpus yothersi*, the endosymbiont *Cardinium* has been associated with female-only colonies. It is unclear what the bacterial composition of *B. yothersi* is, and how common *Cardinium* is in those microbiomes. We performed a comparative analysis of the bacteriomes in three populations of *B. yothersi* and three additional Tetranychoidea species using sequences from V4-fragment of 16S DNA. The bacteriomes were dominated by Bacteroidetes (especially *Cardinium*) and Proteobacteria, showing a remarkably low alpha diversity. *Cardinium* was present in about 22% of all sequences; however, it was not present in *R. indica* and *T. evansi*. In *B. yothersi*, the proportion of *Cardinium* was higher in adults than eggs, suggesting that proliferation of the bacteria could be the result of selective pressures from the host. This hypothesis was further supported because colonies of *B. yothersi* from different populations showed different bacterial assemblages, and bacteriomes from different mite species showed similar abundances of *Cardinium*. A phylogenetic analysis of *Cardinium* revealed that not only specialization but horizontal transmission has been important for this symbiosis. Together, these results represent a glimpse into the evolution of the Tetranychoidea and *Cardinium*.

## 1. Introduction

The mite family Tenuipalpidae has gained prominence in the last two decades as an emerging pest for agricultural and ornamental crops such as citrus, coffee, passion vine, tea, pistachio, and palms [1,2,3]. Particularly, the genus *Brevipalpus* has emerged as a major pest because of its capacity to transmit virus to crop plants. The most prolific vector species that has been reported is *Brevipalpus phoenicis* Geijskes, for which recent studies indicated that the species has been perpetually misidentified, and that it is actually *B. yothersi* Baker [4,5]. This species was reported to transmit viruses associated with two major cytopathology groups [6] and belonging to at least two virus families [7]. Interestingly, these mites reproduce by asexual thelytoky associated with the “feminizing” endosymbiont *Cardinium* [8,9].

Studies have shown that the presence of endosymbiotic bacteria has the potential to manipulate traits such as nutrition [10,11], immune response [12,13], and reproduction [14,15] in arthropods. In fact, it has been proposed that bacterial symbionts have driven to a large degree the evolution of many arthropod taxa by increasing fitness, adaptation, and specialization to different environments [16,17,18]. Such a close relationship between host and endosymbiont is probably the result of both ecological interactions among symbionts and selective pressures posed by the host [19]. Although literature regarding host-symbiont interactions is extensive, less attention has been directed to the study of these interactions under different conditions imposed by different plant hosts.

The ecological conditions of an arthropod host change along different life stages, which also may alter the conditions in the microhabitat of endosymbionts. It has been reported that bacterial communities in larvae of the mosquito *Anopheles gambiae* were different from those in adults: Adult *A. gambiae* showed a decrease in bacterial diversity in comparison to their larvae and there was a drastic shift from a species-rich community to an Enterobacteraceae-dominated bacteriome [20]. Conversely, the bacterial assemblage of the beetle *Agrilus planipennis* was significantly more species-rich in the pre-pupae than in both larval and adult stages [21]. In both cases, a specialization in metabolic function of the bacteriome has been suggested as the consequence (or cause) of these changes. Although ontogenic development in insects is different to that in mites, specialization in bacterial diversity is also expected in mites due to equally significant morphological and physiological changes from eggs to adults.

Specialization of the microbiome has also had effects on processes such as reproduction. For example, *Wolbachia* and *Cardinium* endosymbionts distort sex ratios and affect fitness and survival in a wide variety of arthropods and other invertebrates [8,9,14,16,22,23]. In fact, it has been shown that elimination of *Cardinium* results in decreasing survival and reproductive fitness in insects [24]. If the same situation holds true for *B. yothersi*, it is expected that the vertically-transmitted *Cardinium* will maintain or increase its abundance at different life stages of the host. To the best of our knowledge, no deep assessment of this kind has been performed for the bacterial communities and abundance of *Cardinium* in *B. yothersi*.

We made an assessment of the bacterial communities in populations of *B. yothersi* obtained from different plant hosts and at different life stages. Understanding the microbiota associated to this species will enhance our knowledge about the evolution of its asexuality, and further develop strategies for its management. Specifically, we defined the composition and abundance of different bacterial groups and tested for changes in abundance of the endosymbiont *Cardinium* between mite populations and their adult and egg stages.

## 2. Materials and Methods

### 2.1. Collection, Identification, and Laboratory Rearing of Mite Species

Specimens of *Brevipalpus* mites were initially identified as *B. phoenicis*, but are now recognized as *B. yothersi* Baker [5]. Those specimens were originally collected from three different hosts in Puerto Rico: Sweet orange (*Citrus sinensis*, Rutaceae), Tahiti lime (*Citrus latifolia*, Rutaceae), and glory-bower (*Clerodendrum thomsoniae*, Lamiaceae). Mite colonies were established by a single egg and maintained in fruit-arenas of “Valencia” sweet orange for 10 generations in an environmental chamber (Precision Model 818; Thermo Scientific, Waltham, MA, USA) at 25 °C and 75% relative humidity [25]. As outgroups, all in the Tetranychoidea superfamily, we included samples of adult *Tetranychus evansi* obtained from tomato plants (*Solanum lycopersicum*, Solanaceae) in the field, as well as colonies of *Raoiella indica* from coconut (*Cocos nucifera*, Arecaceae), and an *Oligonychus* mite (from dry beans, *Phaseolus vulgaris*, Fabaceae) collected at the Rio Piedras Agricultural Station. For identification, specimens from all species were slide-mounted in Hoyer’s medium and barcoded using previously described primers for the mitochondrial Cytochrome Oxidase Subunit I gene (COI) [25]. Accession numbers for the mites used in this study were deposited at GenBank (Accession numbers: KP180424 to KP180429).

### 2.2. Genomic DNA Extraction and Pyrosequencing

We extracted total genomic DNA from 20 eggs and 20 mites per colony following the CTAB (Cetyl Trimethyl-Ammonium Bromide) method used in previous studies [25,26]. The DNA extraction manipulations were conducted in a clean laminar flow chamber and we used certified DNA-free tubes and reagents. The mites were washed in 0.05% hypochlorite bleach and rinsed thoroughly with DNA-free water before DNA extraction. We used the primers bac515F (5′-GTGCCAAGCMGCCGCGGTAA-3′) and bac806R (5′-GTGCCAAGCMGCCGCGGTAA-3′) to amplify ≈250 bp of the V4 region coding for part of the 16S Ribosomal Subunit (16S) with the HotStarTaq Plus Master Mix Kit (Qiagen, Hilden, Germany). PCR conditions included an initial stage of 3 min at 94 °C, followed by 28 cycles of 94 °C for 30 s, 53 °C for 40 s, and 72 °C for 1 min. The final elongation step was performed at 72 °C for 5 min. Negative control samples (no mites) were included and processed equally during all steps in order to detect eventual bacterial contamination.

A construct composed by the primers previously mentioned, a linker sequence, and a sample-specific oligonucleotide tag (a.k.a barcode) was incorporated for sequencing on a 454 GS FLX System (Roche, Branford, CT, USA) (Table 1). The inclusion of this linker into the amplicons was made with a second PCR using 1 μL of the previous PCR product under the same conditions. A purification of these PCR products was done with the Agencourt Ampure Beads (Agencourt Bioscience, La Jolla, CA, USA). An estimation of the size and concentration of the amplicons was made by using DNA chips and the Bio-Rad Experion Automated Electrophoresis Station (Bio-Rad, Hercules, CA, USA). The FLX sequencing solution contained 9.6 × 10^6^ DNA molecules/μL mixed with 6 million binding beads. This DNA-bead solution was amplified by using emulsion PCR [27] and then, DNA on the binding beads was denatured with NaOH. An FLX sequencing run was performed using a Genome Sequencer FLX System (Roche) on a 70 × 75 GS Picotiter Plate (PTP) by following instructions from the manufacturer.

### 2.3. Data Processing and Quality Control

We performed an initial processing and filtering of the sequence reads by using a pipeline of scripts included in the MacQIIME 1.8.0 distribution [28]. Sequence (.fasta) and quality (.qual) files were obtained from the flowgram file (.sff) generated by the FLX platform using QIIME’s “process_sff.py” script. During the de-multiplexing, we also eliminated the primer, linkers, and barcodes from the reads. Sequences with average Quality Score lower than 25 were discarded from subsequent analysis. This new set of filtered sequences was denoised and aligned in PyNAST v1.2 against the Greengenes reference alignment [29]. Sequences with less than 30% in similarity to the reference alignment where excluded from subsequent analysis. Identification and elimination of chimeric sequences was performed with ChimeraSlayer v4.29.2010 as implemented in MacQIIME and using the template alignment provided by PyNAST.

### 2.4. Analysis of Bacterial Diversity

We assigned the taxonomy and produced estimations diversity with the pipeline implemented in MacQIIME. Following, we present some of the key points of this pipeline. We made clusters based on a 97% of similarity and identified Operational taxonomic units (OTUs) in the quality-filtered dataset. Each OTU was assigned taxonomy by using the Ribosomal Database Project (RDP) Classifier v2.11 [30] and singletons were discarded from subsequent analyses.

To estimate diversity inside each sample (i.e., alpha diversity) we rarefied the sequences 10 times adding 50 sequences by rarefaction. Averages for the Chao1 and Shannon indexes were calculated based on such rarefactions. Based on the rarefied dataset, we also estimated the unweighted and weighted UniFrac distances, and the Bray-Curtis dissimilarity index. With these estimators, a Principal Coordinate Analysis (PCoA) was performed.

### 2.5. Phylogenetic Analysis of Cardinium Strains

We extracted from the entire dataset the OTUs assigned as *Cardinium* by the RDP Classifier. Additionally, we obtained representative sequences from all the *Cardinium* available at GenBank regardless of the host where it was isolated. Maximum Parsimony trees were created using the algorithm included in MEGA v6 [31]. The resulting consensus tree was resampled 1000 times by bootstrapping and branches with less than 50% in support were collapsed.

## 3. Results

### 3.1. Composition of the Bacteriomes

**Figure 1:**
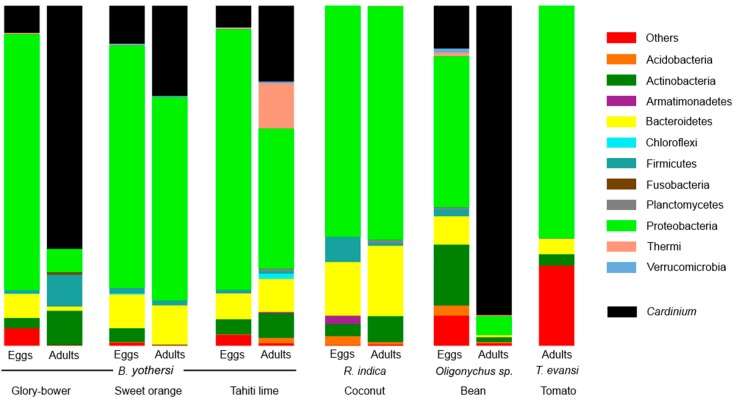
Relative abundance of bacterial phyla found in the mite samples. The proportion of the genus *Cardinium* is shown in black. Singletons were removed from this and subsequent analyses.

Sequences were deposited in GenBank Bioproject PRJNA354805. This is the first assessment of the bacterial communities in *Brevipalpus* and other Tetranychoidea. Our results show that the bacterial communities of those Tetranychoidea were dominated by Bacteroidetes and Proteobacteria. Other groups present in lesser proportions were Actinobacteria, and Firmicutes (Figure 1). Among these, we found OTUs assigned to known bacterial endosymbionts belonging to the genera *Cardinium* (OTUs 33, 190, 233, 314, 335, 445, 455, 457, 460, 547, 625, 805, 843, 888), *Portiera* (OTU 863), *Tremblaya* (OTU 516), and *Wolbachia* (OTU 1043). The most abundant of the endosymbionts was *Cardinium*, being present in about 22% of all sequences; however, it was not present in *R. indica* or *T. evansi* (Table 2). Representative 16S sequences for *Cardinium* and the other endosymbionts are deposited at GenBank (Accession numbers: KX844704 to KX844707). A table with the OTUs for the bacterial taxa found in these mites and their identification numbers is provided as Supplementary Materials (Table S1).

As suggested by the Chao1 index, endosymbionts in adult *Oligonychus* sp. from beans were the least species-rich sample (Chao1 = 11.2), and adult *B. yothersi* from *C. aurantifolia* (Chao1 = 45.6) were the most species-rich sample (Table 3). Nevertheless, the most diverse bacterial community was obtained from *R. indica* from coconut palms (Shannon = 4.2). Overall, *B. yothersi* richness (Chao1 = 31.5) was higher than that of the other mites (Chao1 = 28.1); however, the *B. yothersi* bacteriome was less diverse (Shannon = 2.4) than that of the other species (Shannon = 3.0). When comparing *B. yothersi* eggs with their adults, we observed that increase of *Cardinium* was accompanied by a decrease in the proportion of other OTUs. Specifically, OTUs assigned to Pseudomonadaceae and Burkholderiaceae were proportionally reduced in adults when compared to eggs.

**Figure 2:**
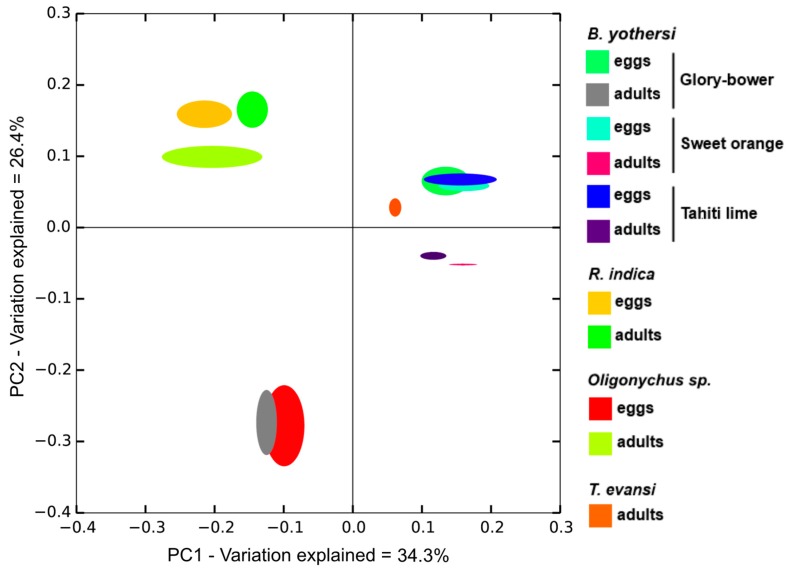
Principal Coordinate Analysis for the dissimilarity among bacterial communities.

In general, the bacterial communities containing OTUs assigned to *Cardinium* were more similar than those lacking the endosymbiont (ANOSIM R_BrayCurtis/UniFrac_ = 0.84/0.59, *p* < 0.05, Figure 2). Specifically, the bacteriomes of the *Oligonychus* mite and *B. yothersi* were more similar among them than to those found in the other mite species. Similarity was higher between the less diverse bacterial communities in the eggs of *B. yothersi*, than to the bacteriomes from their respective adults. As expected, mites where *Cardinium* was the predominant taxa (adult *Oligonychus* sp. and adult *B. yothersi* from *C. thomsoniae*) were almost identical. The bacteriome of *T. evansi* adults was more similar to that of *R. indica* than to other bacteriomes in this study.

### 3.2. Analysis of Cardinium Endosymbionts

The results show that inside each sample of *B. yothersi*, the proportion of OTUs designated as *Cardinium* is higher in adults than in the eggs (Figure 1). In the sample from *C. thomsoniae*, *Cardinium* counts were significantly lower in eggs than in adults (7.9% vs. 71.5%, χ^2^ = 2600.3, *p* < 0.001). For the sample obtained from *C. sinensis*, we obtained the same pattern (11.1% vs. 26.5%, χ^2^ = 322.5, *p* < 0.001), as well for the mites collected originally on *C. aurantifolia* (6.4% vs. 22.2%, χ^2^ = 3164.8, *p* < 0.001). A significant increase in the amount of counts of *Cardinium* sequences were also observed in *Oligonychus* mites obtained from beans. Specifically, *Oligonychus* sp. eggs had 12.6% *Cardinium* sequences whereas the adults had 90.9% (χ^2^ = 546.4, *p* < 0.001).

**Figure 3:**
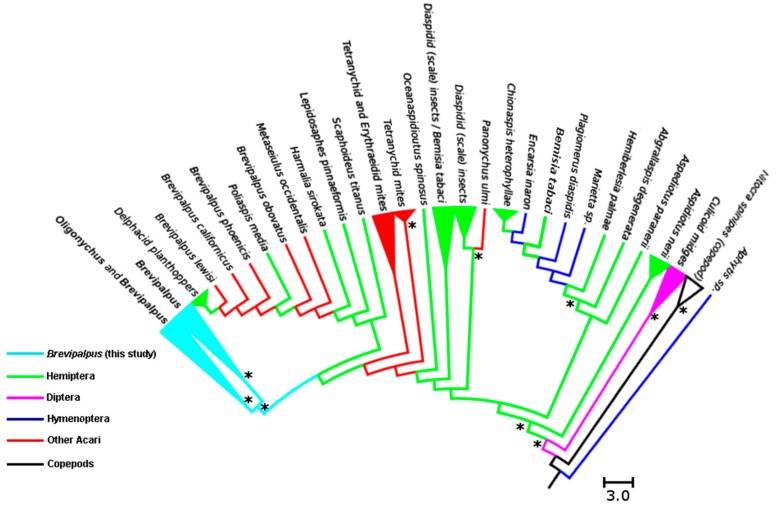
Maximum Parsimony tree for a representative sample of *Cardinium* (microphotograph credit: E.W. Kitajima) sequences from different arthropods available at GenBank, and sequences obtained in this study. Color legend indicates the host from where the *Cardinium* was isolated and sequenced. Asterisks show nodes with support higher than 50% based on bootstrap resampling (1000 replicates).

Our phylogenetic analysis of the sequences from *Cardinium* isolated from different hosts showed that bacteria from Hemiptera, Hymenoptera, and mites form a monophyletic clade. Inside this clade, two main groups can be recognized. One of these groups is dominated by *Cardinium* endosymbionts originating from Hemiptera (whiteflies and scale insects) and some Hymenoptera. The second group contains all the *Cardinium* sequences from this study and isolates from other mites and armored scale insects (Diaspididae: Hemiptera). *Cardinium* from copepods and mosquitoes were placed external to the monophyletic clade, suggesting that these arthropods harbor highly differentiated lineages of these bacteria (Figure 3, Supplementary Materials: Figure S1, Table S2).

## 4. Discussion

### 4.1. Factors Affecting Diversity in Bacterial Communities

The low number of taxa present in the bacterial communities of the mites in this study is concordant with other studies on arthropod bacteriomes [35,36]. Our results support the idea of a “core bacteriome” on animals [37], represented by Proteobacteria, Bacteroidetes, and Actinobateria. The low diversity in arthropod microbiomes, if not an artifact of the analyses, could be attributed to the response from the innate immune system in the hosts [37,38]. In contrast to other animals, the less adaptable immune system in invertebrates would allow only a select combination of bacterial taxa. Even though the immune system may be playing a capital role in shaping the diversity of arthropod microbiomes, there are additional possibilities that we will discuss here.

The fact that bacteriome alpha diversity of these mites is reduced in adults in comparison to eggs reflects the existence of mechanisms that are yet to be described, which are involved in the definition of bacterial assemblages. An increasing number of papers report that competition can be an important driver of reduction of diversity in microbial communities [39,40,41,42]. Most of the studies on arthropod gut microbiomes show that competition may even lead to exclusion of taxa by several mechanisms such as the depletion of nutrients [43] and pH changes [44].

In many cases, such as in these mites, the endosymbiont *Cardinium* increases its relative abundance in the bacterial community possibly by means of competitive exclusion. This would result in a decrease of bacteriome diversity and species richness as has been seen in ticks and fleas [45,46]. However, the host may also shape the bacterial community by generating the conditions for certain bacterial taxa to dominate, a situation known as habitat filtering [47]. In several systems, the dominant endosymbiont confers an advantage in fitness, feeding, or survival to the host [17,24,48]. These advantages conferred by the endosymbiont may evoke responses in the host that ultimately favor their proliferation throughout the life of the host. As our results show, the endosymbiont *Cardinium* was significantly more abundant in adults of *B. yothersi* and *Oligonychus* sp. than in their respective eggs. Similarly, *Sphingomonas* sp. (known endosymbiont in non-arthropod systems [49]) was detected at higher proportions in adults than in eggs of *R. indica*. We were not able to obtain samples from *T. evansi* eggs, but we suspect that *Erwinia* sp. [50] would also be more abundant in the eggs of *T. evansi* than in the adults.

Environmental factors can also influence the composition of microbial communities. The mites in this study come from different hosts and locations that represent the founding bacterial populations. Although *Cardinium* was abundantly present in the three *B. yothersi* colonies, the proportion of *Cardinium* varied among them. The colony from Glory-bower showed a dominance of *Cardinium* over other bacterial taxa, which makes this bacterial community more similar to that found in *Oligonychus* sp. In fleas and ticks that feed on mammals, it has been shown that microbial communities are affected mostly by environmental factors external to the mammal and by mechanisms dictated by the arthropod itself [46,51]. The results in this study are in agreement with the idea that different mite species may converge to have similar bacterial community profiles, and conspecific mites originating from different plant hosts may show different bacterial assemblages.

In this sense, habitat filtering (i.e., original host-plant selection) may be a strong driving force in shaping the bacteriomes of the mites in this study, possibly leading to specialization in the function of these bacterial communities. This filter imposed by the habitat can be seen as the range of conditions and resources that the mite host offers [52]. The host “selects” only a fraction of all the initial diversity, possibly depending upon its physiological requirements. It would also be expected that habitat filtering results in the components of the bacteriome being more phylogenetically related due to host requirements or inability of the symbiont to adapt to different conditions [53,54,55]. In the mites from this study, most of the bacterial species belong to either Bacteroidetes (such as *Cardinium*) or Proteobacteria, which indicates a higher degree of phylogenetic relatedness than would be observed in other communities with lower selective pressures. Nevertheless, this work only represents a snapshot of the existing conditions and under natural, non-controlled habitats, different degrees of competition and habitat filtering may occur depending upon the assessed spatial and temporal scale [56].

### 4.2. Phylogenetic Relationship among Cardinium Strains in Different Systems

The phylogenetic analysis of *Cardinium* strains failed to show convincing evidence that supports phylogenetic correlation between the evolution of these bacteria and their hosts. Previous works on the distribution of *Cardinium* indicated that physiological specialization is noticeable due to phylogenetic clustering of *Cardinium* strains from closely related hosts [57]. Although this idea is not clearly observed in our results, it is possible to infer that *Cardinium* strains show greater phylogenetic relatedness with the *Cardinium* strains present in other mites than to hemipterans and hymenopterans. Additionally, *Cardinium* from copepods and mosquitoes represent highly differentiated lineages from those found in other insects and mites. Although we could not show strong evidence for the idea of the phylogenetic clustering of *Cardinium* and their hosts, our analysis provides evidence about potential horizontal transmission as an important process in the evolution of *Cardinium* and their hosts.

## 5. Conclusions

This study represents the first time that the Next Generation Sequencing approach was used on microbial communities of the agriculturally important Tetranychoidea mites [58]. Our results suggest that the bacteriomes of these mites are relatively low in diversity, as in many other arthropods, although diversity was higher in eggs than in adults. The differences between colonies of *B. yothersi* and similarities among species in regards to the composition of the bacterial assemblages shows that the hosts may be differentially filtering the taxa in their bacteriomes. However, we recognize that competition among bacterial species could also contribute to changes in diversity at different degrees depending upon the assessed temporal and spatial scale. The phylogenetic history of *Cardinium* seems to be driven by physiological specialization of the host and horizontal transmission acting at different degrees. It seems implausible that the asexuality associated with *Cardinium* in arthropods represents an evolutionary dead end [59,60] on the basis that divergence is still extensive at least for the endosymbiont. Understanding the bacteriome associated with the unique biological system represented by *B. yothersi* and other Tetranychoidea is certainly an important step towards enhancing knowledge about the evolution of its asexuality, and might further help to develop strategies for its pest management.

## Figures and Tables

**Table 1:** Specific tags used to identify each of the samples after multiplexed FLX sequencing.

Species	Host Plant	Stage	Tag
*Brevipalpus yothersi*	Glory-bower	eggs	GAGATCAG
		adults	GAGTAGAC
	Sweet orange	eggs	GAGATCTC
		adults	GAGATGAC
	Tahiti lime	eggs	GAGTACTC
		adults	GAGTACAG
*Raoiella indica*	Coconut	eggs	GAGTCACT
		adults	GATGCAAG
*Oligonychus* sp.	Bean	eggs	GATGAGCA
		adults	GATGAGGT
*Tetranychus evansi*	Tomato	adults	GAGTAGTG

**Table 2:** Percentage of sequences per sample classified as mite endosymbionts (according to [32,33,34]).

Mite Species	Host	Stage	*Cardinium*	*Portiera*	*Tremblaya*	*Wolbachia*
*Brevipalpus yothersi*	Glory-bower	Eggs	7.9	-	-	-
		Adults	71.5	-	-	-
	Sweet orange	Eggs	11.1	-	-	0.1
		Adults	26.5	-	-	-
	Tahiti lime	Eggs	6.4	0.3	-	-
		Adults	22.2	-	-	-
*Raoiella indica*	Coconut	Eggs	-	-	-	-
		Adults	-	-	<0.1	-
*Oligonychus* sp.	Bean	Eggs	12.6	-	-	-
		Adults	90.9	-	-	-
*Tetranychus evansi*	Tomato	Adults	-	-	-	-
All the sequences			22.25	0.03	<0.01	<0.01

**Table 3:** Average estimations of bacteria diversity (±standard deviation) based on rarefaction of samples.

Species	Host Plant	Stage	Chao1	Shannon
*Brevipalpus yothersi*	Glory-bower	eggs	100.88 ± 16.95	3.32 ± 0.13
		adults	94.75 ± 16.26	2.79 ± 0.09
	Sweet orange	eggs	95.20 ± 11.62	2.73 ± 0.09
		adults	55.93 ± 9.46	2.12 ± 0.07
	Tahiti lime	eggs	97.66 ± 18.88	2.36 ± 0.10
		adults	146.02 ± 21.25	4.24 ± 0.09
*Raoiella indica*	Coconut	eggs	82.31 ± 15.13	4.41 ± 0.09
		adults	115.49 ± 15.59	4.74 ± 0.10
*Oligonychus* sp.	Bean	eggs	79.46 ± 7.19	4.42 ± 0.09
		adults	47.17 ± 13.36	1.00 ± 0.07
*Tetranychus evansi*	Tomato	adults	54.52 ± 4.45	3.66 ± 0.05

## Supplementary Materials

The following are available online at www.mdpi.com/2075-4450/7/4/80/s1. Figure S1: Detailed Maximum Parsimony for *Cardinium* sequences from this study and others, Table S1: OTU table (contains the counts and taxonomic assignation for each OUT in this study), Table S2: Sequences from GenBank published elsewhere that were used in this study for phylogenetic inference in *Cardinium*.
