# Supplementary material for: Reduced Diversity in the Bacteriome of the Phytophagous Mite Brevipalpus yothersi (Acari: Tenuipalpidae)
Source: Insects. 2016 Dec 20;7(4):80. doi: 10.3390/insects7040080 (PMC5198228; doi:10.3390/insects7040080)
Supplement: Supplementary file 1 [file insects-07-00080-s001.zip › insects-155462-supplementary/insects-155462-supplementary.pdf]

Supplementary Materials: Reduced Diversity in the Bacteriome of the Phytophagous Mite *Brevipalpus yothersi* (Acari: Tenuipalpidae)

Oscar E. Ospina, Steven E. Massey and Jose Carlos Verle Rodrigues

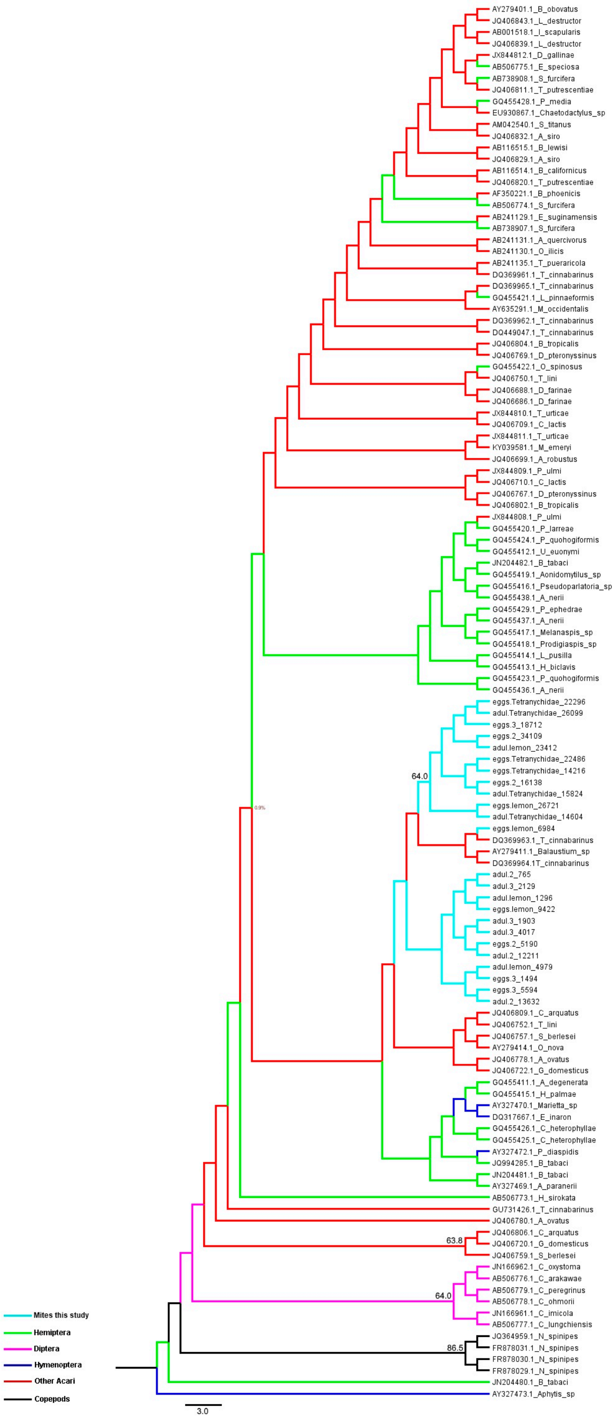

Figure S1. Complete version of the Maximum Parsimony tree for a representative sample of *Cardinium* sequences from different arthropods available at GenBank, and sequences obtained in this study. Color legend indicates the host from where the *Cardinium* were isolated and sequenced. Support values based on bootstrap resampling (1000 replicates) that were higher than 50% are shown. See Table S2 for accession numbers in the tree.

Table S2. Sequences from GenBank published elsewhere that were used in this study for phylogenetic inference in *Cardinium*.

Accession number	Host	Reference
GQ455411	*Abgrallaspis degenerata*	--
AB241131	*Amphitetranychus quercivorus*	--
GQ455419	*Aonidomytilus* sp.	--
AY327473	*Aphytis* sp.	[1]
AY327469	*Aspediotus paranerii*	[1]
GQ455436<br>GQ455437<br>GQ455438	*Aspidiotus nerii*	--
AY279411	*Balaustium* sp.	[2]
JQ994285<br>JN204480<br>JN204481<br>JN204482	*Bemisia tabaci*	--<br>[3]
AB116514	*Brevipalpus californicus*	--
AB116515	*Brevipalpus lewisi*	--
AY279401	*Brevipalpus obovatus*	[2]
AF350221	*Brevipalpus phoenicis*	--
GQ455425<br>GQ455426	*Chionaspis heterophyllae*	--
AB506776	*Culicoides arakawae*	[4]
JN166961	*Culicoides imicola*	[5]
AB506777	*Culicoides lungchiensis*	[4]
AB506778	*Culicoides ohmorii*	[4]
JN166962	*Culicoides oxystoma*	[5]
AB506779	*Culicoides peregrinus*	[4]
DQ317667	*Encarsia inaron*	[6]
AB241129	*Eotetranychus suginamensis*	--
AB506775	*Euides speciosa*	[4]
AB506773	*Harmalia sirokata*	[4]
GQ455415	*Hemiberlesia palmae*	--
GQ455413	*Howardia biclavis*	--
GQ455421	*Lepidosaphes pinnaeformis*	--
GQ455414	*Leucaspis pusilla*	--
AY327470	*Marietta* sp.	[1]
GQ455417	*Melanaspis* sp.	--
AY635291	*Metaseiulus occidentalis*	[7]
FR878029<br>FR878030<br>FR878031<br>JQ364959	*Nitocra spinipes*	--<br>[8]
GQ455422	*Oceanaspidiotus spinosus*	--
GQ455423<br>GQ455424	*Palinaspis quohogiformis*	--
GQ455429	*Pallulaspis ephedrae*	--
JX844808<br>JX844809	*Panonychus ulmi*	--
AY327472	*Plagiomerus diaspidis*	[1]
GQ455428	*Poliaspis media*	--
GQ455418	*Prodigisapis* sp.	--

GQ455420	*Protargionia larreae*	--
GQ455416	*Pseudoparlatoria* sp.	--
AM042540	*Scaphoideus titanus*	[9]
AB506774	*Sogatella furcifera*	[4]
DQ369965		[10]
DQ449047	*Tetranychus cinnabarinus*	
GU731426		--
AB241135	*Tetranychus pueraricola*	--
JX844811	*Tetranychus urticae*	--
GQ455412	*Unaspis euonymi*	--

References

1. Zchori-Fein, E.; Perlman, S.J. Distribution of the bacterial symbiont *Cardinium* in arthropods. *Mol. Ecol.* **2004**, *13*, 2009–2016.
2. Weeks, A.R.; Velten, R.; Stouthamer, R. Incidence of a new sex-ratio-distorting endosymbiotic bacterium among arthropods. *Proc. R. Soc. B Biol. Sci.* **2003**, *270*, 1857–1865.
3. Singh, S.T.; Priya, N.G.; Kumar, J.; Rana, V.S.; Ellango, R.; Joshi, A.; Priyadarshini, G.; Asokan, R.; Rajagopal, R. Diversity and phylogenetic analysis of endosymbiotic bacteria from field caught *Bemisia tabaci* from different locations of North India based on 16S rDNA library screening. *Infect. Genet. Evol.* **2012**, *12*, 411–419.
4. Nakamura, Y.; Kawai, S.; Yukuhiro, F.; Ito, S.; Gotoh, T.; Kisimoto, R.; Yanase, T.; Matsumoto, Y.; Kageyama, D.; Noda, H. Prevalence of *Cardinium* bacteria in planthoppers and spider mites and taxonomic revision of "Candidatus *Cardinium hertigii*" based on detection of a new *Cardinium* group from biting midges. *Appl. Environ. Microbiol.* **2009**, *75*, 6757–6763.
5. Morag, N.; Klement, E.; Saroya, Y.; Lensky, I.; Gottlieb, Y. Prevalence of the symbiont *Cardinium* in Culicoides (Diptera: Ceratopogonidae) vector species is associated with land surface temperature. *FASEB J.* **2012**, *26*, 4025–4034.
6. Perlman, S.J.; Kelly, S.E.; Zchori-Fein, E.; Hunter, M.S. Cytoplasmic incompatibility and multiple symbiont infection in the ash whitefly parasitoid, *Encarsia inaron*. *Biol. Control* **2006**, *39*, 474–480.
7. Jeyaprakash, A.; Hoy, M.A. Multiple displacement amplification in combination with high-fidelity PCR improves detection of bacteria from single females or eggs of *Metaseiulus occidentalis* (Nesbitt) (Acari: Phytoseiidae). *J. Invertebr. Pathol.* **2004**, *86*, 111–116.
8. Edlund, A.; Ek, K.; Breitholtz, M.; Gorokhova, E. Antibiotic-induced change of bacterial communities associated with the copepod *Nitocra spinipes*. *PLoS One* **2012**, *7*, e33107.
9. Marzorati, M.; Alma, A.; Sacchi, L.; Pajoro, M.; Palermo, S.; Brusetti, L.; Raddadi, N.; Balloi, A.; Tedeschi, R.; Clementi, E.; Corona, S.; Quaglino, F.; Bianco, P.A.; Beninati, T.; Bandi, C.; Daffonchio, D. A novel Bacteroidetes symbiont is localized in *Scaphoideus titanus*, the insect vector of Flavescence Doree in *Vitis vinifera*. *Appl. Environ. Microbiol.* **2006**, *72*, 1467–1475.
10. Liu, Y.; Miao, H.; Hong, X.Y. Distribution of the endosymbiotic bacterium *Cardinium* in chinese populations of the carmine spider mite *Tetranychus cinnabarinus* (Acari: Tetranychidae). *J. Appl. Entomol.* **2006**, *130*, 523–529.

© 2016 by the authors; licensee MDPI, Basel, Switzerland. This article is an open access article distributed under the terms and conditions of the Creative Commons by Attribution (CC-BY) license (http://creativecommons.org/licenses/by/4.0/).
